# A critical period for experience-dependent development of the feelings of safety during early infancy: A polyvagal perspective on anger and psychometric tools to assess perceived safety

**DOI:** 10.3389/fnint.2022.915170

**Published:** 2022-07-18

**Authors:** Andrea Poli, Angelo Gemignani, Carlo Chiorri, Mario Miccoli

**Affiliations:** ^1^Department of Clinical and Experimental Medicine, University of Pisa, Pisa, Italy; ^2^Department of Surgical, Medical and Molecular Pathology and of Critical Care Medicine, University of Pisa, Pisa, Italy; ^3^Department of Educational Sciences, University of Genoa, Genoa, Italy

**Keywords:** anger, interpersonal violence, polyvagal theory, hierarchical emotional response, vagus nerve, myelination, development

## Introduction

Due to its distinct and widely recognizable pattern of face expression, anger has always been included in the repertoire of basic emotions (Ekman, 1999). Relying on polyvagal theory, Beauchaine et al. (2007) summarized the results of three studies (Beauchaine, 2001; Mead et al., 2004; Crowell et al., 2006) evaluating autonomic nervous system functioning in children manifesting aggression and conduct problems, aged 4–18. Children with aggressive oppositional defiant disorder or conduct disorder exhibited both sympathetic hypo-arousal at baseline and sympathetic insensitivity to reward at a very early age, marking a general disinhibitory tendency. In addition to this disinhibition, PNS deficiencies were found and contributed to increased emotional lability. Using transcutaneous vagus nerve stimulation (tVNS), Steenbergen et al. (2021), investigating subjects with age ranging from 18 to 28, found that active tVNS, compared to sham stimulation, enhanced the recognition of anger but reduced the ability to recognize sadness.

According to developmental research, an actual expression of anger does not emerge until the last months of the first year of life (Sroufe, 1996). According to this, research on 5-, 12-, and 15-month-old infants has shown that an adult-like, late, non-linear pattern of cortical response to masked faces at various levels of visibility emerged as early as 5 months of age, starting around 900 ms, possibly due to the development of the right fusiform gyrus (Guy et al., 2016) and its increased sensitivity to fearful faces from 5 to 12 months (Xie et al., 2019; Chen et al., 2021). Subsequently, this late component shifted to a more sustained and faster response in older infants (~750 ms), to reach around 300 ms in adults (Kouider et al., 2013). Consequently, in infants aged 5–12 months exposed to facial expressions of happiness, fear, and anger with normal levels of visibility, the N290 event-related potential (ERP) component was found to be larger in amplitude in response to angry and happy faces than to angry ones, revealing greater cortical activation in the right fusiform face area, while the P400 and the negative-central (Nc) ERP components were found to be larger in amplitude in response to angry faces than to happy and fearful ones, revealing greater activation of the posterior cingulate cortex (PCC)/precuneus associated with the allocation of infants' attention. Interestingly, these effects emerged at 5 months, became well established at 7 months, and then disappeared at 12 months (Xie et al., 2019; Chen et al., 2021). As extensively shown for sensory development (Berardi et al., 2003; Hübener and Bonhoeffer, 2014; Ribot et al., 2021), this evidence may suggest a sensitive period for emotional development (Woodard and Pollak, 2020) related, in particular, to the learning of safety (Porges, 2015, 2022).

## Hierarchical emotional responses: A polyvagal perspective on anger

It is possibly throughout the process of the learning of safety that newborns may become aware that their intentional actions can actually be blocked by internal or external factors, so a definite anger reaction may emerge (Williams, 2017). Internal factors, such as self-disgust, self-criticism (Zahn et al., 2015), and shame proneness have been found to be associated with anger suppression and conflict avoidance (Cheung and Park, 2010) as well as with subsequent anger outbursts (Alia-Klein et al., 2020). In addition, structural equation modeling showed that self-criticism predicted shame and guilt, which, in turn, predicted problematic dysfunctional outcomes such as addiction and problematic behavior (Sassover et al., 2021; Snoek et al., 2021). It has been proposed that anger may be an emotion supporting goal-directed behavior when an environmental situation prevents the desired goal to be fulfilled, causing frustration (Panksepp, 2005). In addition, anger may function as a protection from an external predatory attack. In this case, anger may emerge as a possible consequence of fear (Wilkowski and Robinson, 2010).

According to the polyvagal theory, the structural organization and function of the human autonomic nervous system are hierarchically based on its phylogenetic heritage (Porges, 2007, 2009, 2022). The myelinated ventral vagal complex (VVC), whose cardioinhibitory fibers emerge from the nucleus ambiguus (NA) in the brainstem, is the source of the social engagement system (SES). Due to its myelinated fibers, the VVC is the least homeostatically disruptive, phylogenetically youngest, and fastest responding challenge-response system. The sympathetic nervous system (SNS) is phylogenetically older than the VVC, and its activation promotes increased heart rate, breathing, and mobilized behaviors in order to implement active responses to threats such as escape or defensive confrontation. The Dorsal Vagal Complex (DVC) is the phylogenetically oldest autonomic subsystem, with unmyelinated cardioinhibitory fibers originating in the dorsal motor nucleus (DMNX) of the vagus in the brainstem. It also has a vestigial immobilization function that initially emerged in early vertebrates (Porges, 2021a). The DVC innervates organs below the diaphragm and is involved in both homeostatic and threat reactions. When recruited during threat responses, this complex also disrupts digestion and conserves metabolic resources (Porges, 2007, 2009). Interestingly, the DVC is also the system that is primarily involved in post-traumatic responses after psychological trauma (Kolacz and Porges, 2018; Kolacz et al., 2019).

Polyvagal theory posits that the evolution of the mammalian social engagement system (SES) was aided by the integration of the myelinated cardiac vagal pathways with neural regulation of the face and head. Early atypical coordination of this system is a predictor of following social behavior and emotional control issues. The brain evaluation of environmental danger determines the preferential recruitability of the SES or the sequential hierarchical recruitment of the SNS or the DVC. According to polyvagal theory, the neural evaluation of risk is achieved through neuroception, a neural reflexive mechanism, which is distinct from perception and is capable of instantly shifting the physiological state and of distinguishing safe, dangerous, or life-threatening environmental and visceral features. A neuroception of safety supports the SES in safe contexts, and the autonomic state is adaptively adjusted to decrease SNS activity and protect the oxygen-dependent central nervous system, particularly the cortex, from the metabolically conservative reactions of the DVC (e.g., fainting). Alternatively, a neuroception of danger, or life threat, favors the recruitment of SNS or DVC (Porges, 2003, 2007, 2009, 2021b).

Interestingly, recent evidence investigating the maturation of cardiac autonomic nervous system activity in children and adolescents showed that the cardiac parasympathetic nervous system (PNS) and SNS activity in childhood follow different maturational trajectories. In particular, in the first months of life, an increase in heart rate variability (HRV) and in the number of myelinated fibers in the vagus nerve has been reported. Specifically, HRV increases steadily from the first months to early and middle childhood, then plateaus in late childhood/early adolescence (~age 11) and does not fluctuate or decrease during adolescence, while respiratory sinus arrhythmia (RSA) peaked between 7 and 8 years old. Regarding SNS development, it was found that the mean pre-ejection period showed a distinct maturational effect, as it increases linearly from early childhood to late adolescence, thus implying that SNS activity declines with age. Overall, these results suggest that PNS activity increases rapidly after birth, plateaus in middle childhood, and then declines at the end of adolescence, while SNS shows a steady decline over time (Harteveld et al., 2021). More specifically, the developmental pattern of myelinated and unmyelinated fibers of the human vagus nerve in term infants has long been known. A significant increase was found in the average content of myelin in myelinated fibers, indicating active myelination during the first 9 months of life without a significant change in fiber axonal properties. The density of myelinated fibers increases with age, both in terms of total quantity and in respect to unmyelinated fibers, implying a steady transition from unmyelinated to myelinated axons during the first year of life, notably during the first 3 months (Pereyra et al., 1992). Furthermore, in victims of sudden infant death syndrome (SIDS) between 1 and 9 months of age, more small and fewer large myelinated vagal fibers were detected, compared to unmyelinated fibers, in SIDS than in controls, implying an immature condition of the vagus nerve in SIDS (Becker et al., 1993).

## A critical period for experience-dependent development of the feelings of safety during early infancy

**Figure 1:**
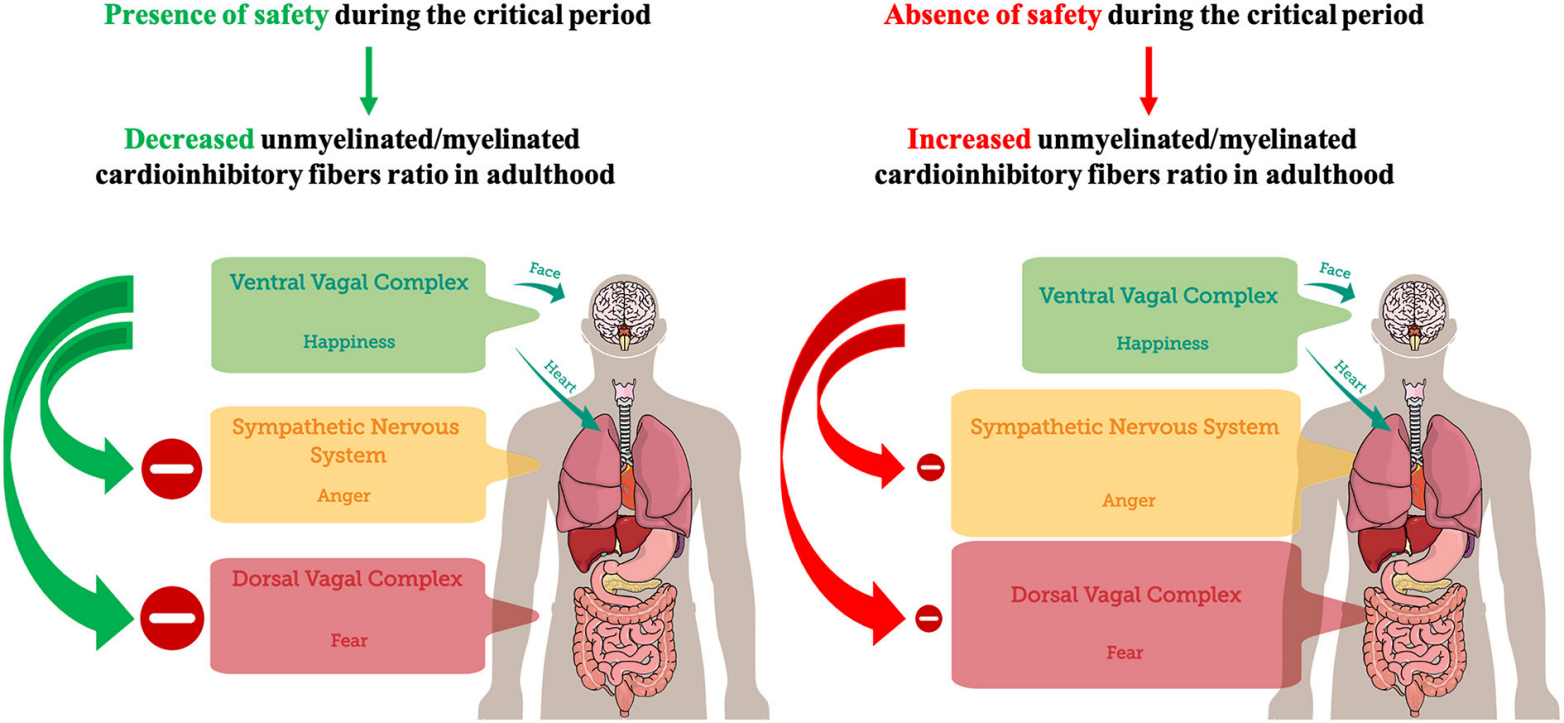
A postnatal critical period for the development of feelings of safety. The presence of safety during the critical period (the first year of life) may lead to a decreased unmyelinated/myelinated cardioinhibitory fibers ratio in adulthood, promoting VVC development, adaptive inhibition of SNS and DVC, and emotional self-regulation. Conversely, the absence of safety during the critical period may lead to an increased unmyelinated/myelinated cardioinhibitory fibers ratio in adulthood, dampening VVC development. Dampened VVC activity reduces the capacity of adaptive inhibition of SNS and DVC, and emotional self-regulation. Hence, environmental detection of unsafety cues may preferentially trigger SNS-mediated anger in order to avoid DVC-mediated immobilization with fear.

Taken together, the results reviewed in this work indicate that at birth, regarding PNS, an increased unmyelinated-to-myelinated fibers ratio (UMR) may be prevalent, suggesting an increased probability of DVC states. Therefore, the subsequent need to experience a neuroception of safety for the infant may be the neurophysiological underpinning of human attachment (Feldman, 2017). Experiencing safety can promote the maturation of vagus' myelinated fibers and the transition from unmyelinated to myelinated axons, decreasing UMR, during the first year of life as a critical period (Woodard and Pollak, 2020). Accordingly, considering the increased UMR of the PNS during the first 3 months of life (Pereyra et al., 1992), it could be hypothesized that it may represent a sensitive period for early environmental safety detection through the DVC, in order to possibly establish a baseline threshold for threat detection and for the emergence of fear. Possibly, contingent experience of safety may promote fiber myelination, the decrease of UMR of PNS, and the emergence of VVC, through a process of activity-dependent myelination (Fields, 2015; Noori et al., 2020) that may operate during the critical period, but also throughout life (Faria et al., 2019). On the contrary, experiencing unsafety may prevent the decrease of UMR of PNS, potentially promoting SIDS (Becker et al., 1993), and the linear age-dependent increase in PEP, which allows for a decrease in the cardiac effects of SNS activity with age (Harteveld et al., 2021). Therefore, in adulthood, after closure of the critical period, unsafety detection in the environment may preferentially promote the emergence of mobilizing sympathetic self-protective strategies related to anger to avoid the prevalence of immobilizing DVC self-protective strategies related to fear (Figure 1). Interestingly, patients with panic disorder were characterized by relative reactive sympathetic dominance in response to mental stress compared to healthy controls (Kotianova et al., 2018).

Consequently, the right fusiform face area, related to the N290 ERP component, found to be larger in amplitude in response to fearful and happy faces than to angry faces (Xie et al., 2019), has also been found to be associated with HRV and PNS (Critchley et al., 2003). Therefore, if safety is detected, the activity of myelinated VVC and the emotion of happiness can be promoted; conversely, if unsafety is detected the activity of unmyelinated DVC and the emotion of fear can be promoted. In order to avoid the immediate predominance of immobilizing DVC self-protective strategies, anger may be recruited right after unsafety detection. Consistent with this, the PCC/precuneus were related to the P400 and the Nc ERP components, and found to be larger in response to angry faces than to happy and fearful faces (Xie et al., 2019). Furthermore, PCC/precuneus, which are brain areas belonging to the default mode network that is typically associated with parasympathetic functions (Beissner et al., 2013), were found to be significantly involved in SNS regulation (Taylor et al., 2016). This finding may explain the evidence related to the reciprocally coupled activation of PNS and SNS during both rest and acute stress (Weissman and Mendes, 2021). Interestingly, individuals with higher baseline RSA, an index of VVC, showed more reciprocal coupling than those with lower baseline RSA, reflecting greater coordination of physiological response (Weissman and Mendes, 2021). Furthermore, the disappearance of these effects at 12 months (Xie et al., 2019), possibly upon completion of UMR increase (Pereyra et al., 1992), may implement what has been learnt during the critical period for safety learning in the instinctively coupled activation of PNS and SNS. In accordance with these observations, regarding interpersonal violence it was found that perpetrators of intimate partner violence showed higher heart rate and skin conductance levels, shorter PEP, and lower RSA values during recovery from stress, as well as more anger and worse mood after stress (Romero-Martínez et al., 2021). Furthermore, individuals who exhibited longer cardiac PEP at rest and reduced PEP reactivity scored higher on measures of behavioral problems and aggression, while individuals who exhibited lower baseline RSA and greater RSA withdrawal scored lower on prosocial behavior and emotion regulation (Beauchaine et al., 2013). Consequently, RSA has been proposed as a transdiagnostic biomarker of emotion regulation and psychopathology (Beauchaine, 2015; Beauchaine and Bell, 2020).

## Neurophysiologically-informed psychometric tools to assess perceived safety and future directions

Since traumatic symptoms arise from unregulated threat preoccupation, when self-regulation is not available (Motsan et al., 2021), assessing safety through the use of psychometric tools that show sound psychometric properties, and that have been rooted in the organization of the autonomic nervous system, is recommended. Considering the importance of safety evaluation within a therapeutic context, recent research has developed new tools to assess feelings of safety (see, e.g., Morton et al., 2021; Poli et al., 2021.

Future research should focus on interventions promoting safety. Maternal-newborn skin-to-skin contact may be an early essential factor needed to promote the development of the feelings of safety and the organization of the infant's physiological systems, including stress reactivity, autonomic functioning, and sleep patterns (Feldman et al., 2002). Skin-to-skin contact was able to increase autonomic functioning (measured by RSA) and enhanced child cognitive development and executive functions from 6 months to 10 years. By 10 years of age, children receiving skin-to-skin contact showed attenuated stress response, improved RSA, organized sleep, and better cognitive control (Feldman et al., 2014). In accordance with this, regarding medial prefrontal cortex (mPFC), in mice it has been shown that chronic maternal separation (MS) during the first 2 weeks of postnatal life led to a depletion of the oligodendrocyte progenitor pool in MS adults that was associated to pro-depressive effects and short-term memory impairment. Conversely, chemogenetic neuronal activation normalized oligodendrocyte progenitor pool in MS animals prevented the pro-depressive effects and short-term memory impairment of MS, identifying neuronal activity as a critical intervention for early-life stress and to promote mPFC-related behaviors (Teissier et al., 2020).

## Author contributions

AP: conceptualization and writing—original draft preparation. AP and MM: methodology, validation, investigation, resources, data curation, supervision, and project administration. AP, AG, CC, and MM: writing—review and editing. All authors have read and agreed to the published version of the manuscript.

## Conflict of interest

The authors declare that the research was conducted in the absence of any commercial or financial relationships that could be construed as a potential conflict of interest.

## Publisher's note

All claims expressed in this article are solely those of the authors and do not necessarily represent those of their affiliated organizations, or those of the publisher, the editors and the reviewers. Any product that may be evaluated in this article, or claim that may be made by its manufacturer, is not guaranteed or endorsed by the publisher.
